# The association between the neutrophil-to-lymphocyte ratio, platelet-to-lymphocyte ratio, and monocyte-to-lymphocyte ratio and systemic sclerosis and its complications: a systematic review and meta-analysis

**DOI:** 10.3389/fimmu.2024.1395993

**Published:** 2024-05-10

**Authors:** Angelo Zinellu, Arduino A. Mangoni

**Affiliations:** ^1^ Department of Biomedical Sciences, University of Sassari, Sassari, Italy; ^2^ Discipline of Clinical Pharmacology, College of Medicine and Public Health, Flinders University, Bedford Park, SA, Australia; ^3^ Department of Clinical Pharmacology, Flinders Medical Centre, Southern Adelaide Local Health Network, Bedford Park, SA, Australia

**Keywords:** neutrophil-to-lymphocyte ratio, platelet-to-lymphocyte ratio, monocyte-to-lymphocyte ratio, systemic sclerosis, disease complications, biomarkers, inflammation

## Abstract

**Introduction:**

The identification of new, easily measurable biomarkers might assist clinicians in diagnosing and managing systemic sclerosis (SSc). Although the full blood count is routinely assessed in the evaluation of SSc, the diagnostic utility of specific cell-derived inflammatory indices, i.e., neutrophil-to-lymphocyte ratio (NLR), platelet-to-lymphocyte ratio (PLR), and monocyte-to-lymphocyte ratio (MLR), has not been critically appraised in this patient group.

**Methods:**

We conducted a systematic review and meta-analysis of studies investigating the NLR, PLR, and MLR, in SSc patients and healthy controls and in SSc patients with and without relevant complications. PubMed, Scopus, and Web of Science were searched from inception to 23 February 2024. Risk of bias and certainty of evidence were assessed using validated tools.

**Results:**

In 10 eligible studies, compared to controls, patients with SSc had significantly higher NLR (standard mean difference, SMD=0.68, 95% CI 0.46 to 0.91, p<0.001; I^2^ = 74.5%, p<0.001), and PLR values (SMD=0.52, 95% CI 0.21 to 0.83, p=0.001; I^2^ = 77.0%, p=0.005), and a trend towards higher MLR values (SMD=0.60, 95% CI -0.04 to 1.23, p=0.066; I^2^ = 94.1%, p<0.001). When compared to SSc patients without complications, the NLR was significantly higher in SSc with interstitial lung disease (ILD, SMD=0.31, 95% CI 0.15 to 0.46, p<0.001; I^2^ = 43.9%, p=0.11), pulmonary arterial hypertension (PAH, SMD=1.59, 95% CI 0.04 to 3.1, p=0.045; I^2^ = 87.6%, p<0.001), and digital ulcers (DU, SMD=0.43, 95% CI 0.13 to 0.74, p=0.006; I^2^ = 0.0%, p=0.49). The PLR was significantly higher in SSc patients with ILD (SMD=0.42, 95% CI 0.25 to 0.59, p<0.001; I^2^ = 24.8%, p=0.26). The MLR was significantly higher in SSc patients with PAH (SMD=0.63, 95% CI 0.17 to 1.08, p=0.007; I^2^ = 66.0%, p=0.086), and there was a trend towards a higher MLR in SSc patients with ILD (SMD=0.60, 95% CI -0.04 to 1.23, p=0.066; I^2^ = 94.1%, p<0.001).

**Discussion:**

Pending the results of appropriately designed prospective studies, the results of this systematic review and meta-analysis suggest that blood cell-derived indices of inflammation, particularly the NLR and PLR, may be useful in the diagnosis of SSc and specific complications.

**Systematic review registration:**

https://www.crd.york.ac.uk/PROSPERO/, identifier CRD42024520040.

## Introduction

Systemic sclerosis (SSc) is a chronic autoimmune condition that affects primarily women and is characterized by progressive fibrosis of the skin and various organs and systems as well as vascular dysfunction (1, 2). The estimated incidence and prevalence of SSc globally are between 8 and 56 new cases per million persons per year and between 38 and 341 cases per million persons, respectively (3). The diagnosis and overall clinical evaluation of patients with SSc is based on specific findings on physical examination and serological abnormalities (4). Such abnormalities include a positive antinuclear antibody (5), anti-topoisomerase I antibody (6), anticentromere antibody (7), anti-RNA polymerase III antibody (8), and antibodies to Th/To (9). However, the diagnosis of SSc is not always straightforward given the overlap with other autoimmune conditions, particularly in the early stages of the disease (10–12). These challenges have prompted the search for novel biomarkers of SSc and its complications, e.g., interstitial lung disease (ILD), pulmonary arterial hypertension (PAH), and digital ulcers (DU), to enhance diagnosis and management (13–17).

An emerging set of inflammatory biomarkers in various autoimmune disorders is represented by specific indices derived from blood cell types that are routinely assessed as part of a full blood count. These cell types, particularly neutrophils, platelets, lymphocytes, and monocytes, have been shown to play an important role in the pathophysiology of SSc in experimental and clinical studies (18–27). A relatively higher neutrophil-to-lymphocyte ratio (NLR), platelet-to-lymphocyte ratio (PLR), and monocyte-to-lymphocyte ratio (MLR) have been shown to successfully discriminate between the presence and the absence of specific autoimmune conditions as well as the presence and absence of active disease in those affected, e.g., rheumatoid arthritis (28–30), psoriasis (31, 32), and systemic lupus erythematosus (33–35). However, the potential diagnostic role of the NLR, PLR, and MLR in SSc and relevant complications has not been critically appraised.

Therefore, we sought to address this issue by conducting a systematic review and meta-analysis of studies investigating the NLR, PLR, and MLR in patients with SSc and healthy controls and in SSc patients with and without specific complications. We hypothesized that the NLR, PLR, and MLR were significantly higher in SSc patients vs. controls and in SSc patients with complications vs. SSc patients without. Where possible, we conducted meta-regression and subgroup analyses to investigate possible associations between the effect size of the between-group differences in these hematological cell indices and pre-defined study and patient characteristics.

## Materials and methods

### Search strategy and study selection

We conducted a systematic search for relevant publications in the electronic databases PubMed, Scopus, and Web of Science, from inception to 23 February 2024, using the following terms: “systemic sclerosis” OR “scleroderma” OR “SSc” and “neutrophil to lymphocyte ratio” OR “neutrophil-to-lymphocyte ratio” OR “NLR” OR “platelet to lymphocyte ratio” OR “platelet-to-lymphocyte ratio” OR “PLR” OR “monocyte-to-lymphocyte ratio” OR “monocyte to lymphocyte ratio” OR “MLR”. Two investigators independently performed a review of the abstracts and full text of the publications based on pre-specified inclusion criteria: (i) the investigation of the NLR and/or PLR and/or MLR in patients with SSc diagnosed according accepted guidelines and healthy controls in a case-control study, (ii) the assessment of the NLR and/or PLR and/or MLR in SSc patients with or without specific complications in a case-control study, (iii) the recruitment of adult participants, and (iv) the availability of the full text of the publication in English language. The two investigators also hand searched the references of individual publications to identify additional studies.

The two investigators independently extracted the following variables from each article: year of publication, first author, study design, country where the study was conducted, sample size, age, male to female ratio, NLR, PLR, and MLR values, and presence of relevant complications.

We assessed the risk of bias, the certainty of evidence, and presence of publication bias using validated tools (36–40). We fully adhered to the Preferred Reporting Items for Systematic reviews and Meta-Analyses (PRISMA) 2020 statement (**Supplementary Table 1**) (41), and registered the study protocol in an official repository (PROSPERO registration number: CRDCRD42024520040).

### Statistical analysis

We calculated standardized mean differences (SMDs) and 95% confidence intervals (CIs) and created forest plots to assess possible differences in NLR, PLR, and MLR value between SSc patients and healthy controls and between SSc patients with and without complications (p-value for significance set at <0.05). Data transformations to obtain means and standard deviations from medians and interquartile ranges or medians and ranges were performed using established methods (42). Heterogeneity of the SMD was assessed using the Q-statistic (significance level at p<0.10). A random-effect model was used for meta-analyses with high heterogeneity (43, 44). Sensitivity analysis was assessed using conventional methods (45).

Meta-regression and subgroup analyses were conducted to assess possible associations between the effect size and study design, study country, age, male to female ratio, and presence of complications. We used Stata 14 for all statistical analyses (Stata Corp., College Station, TX, USA).

## Results

**Figure 1:**
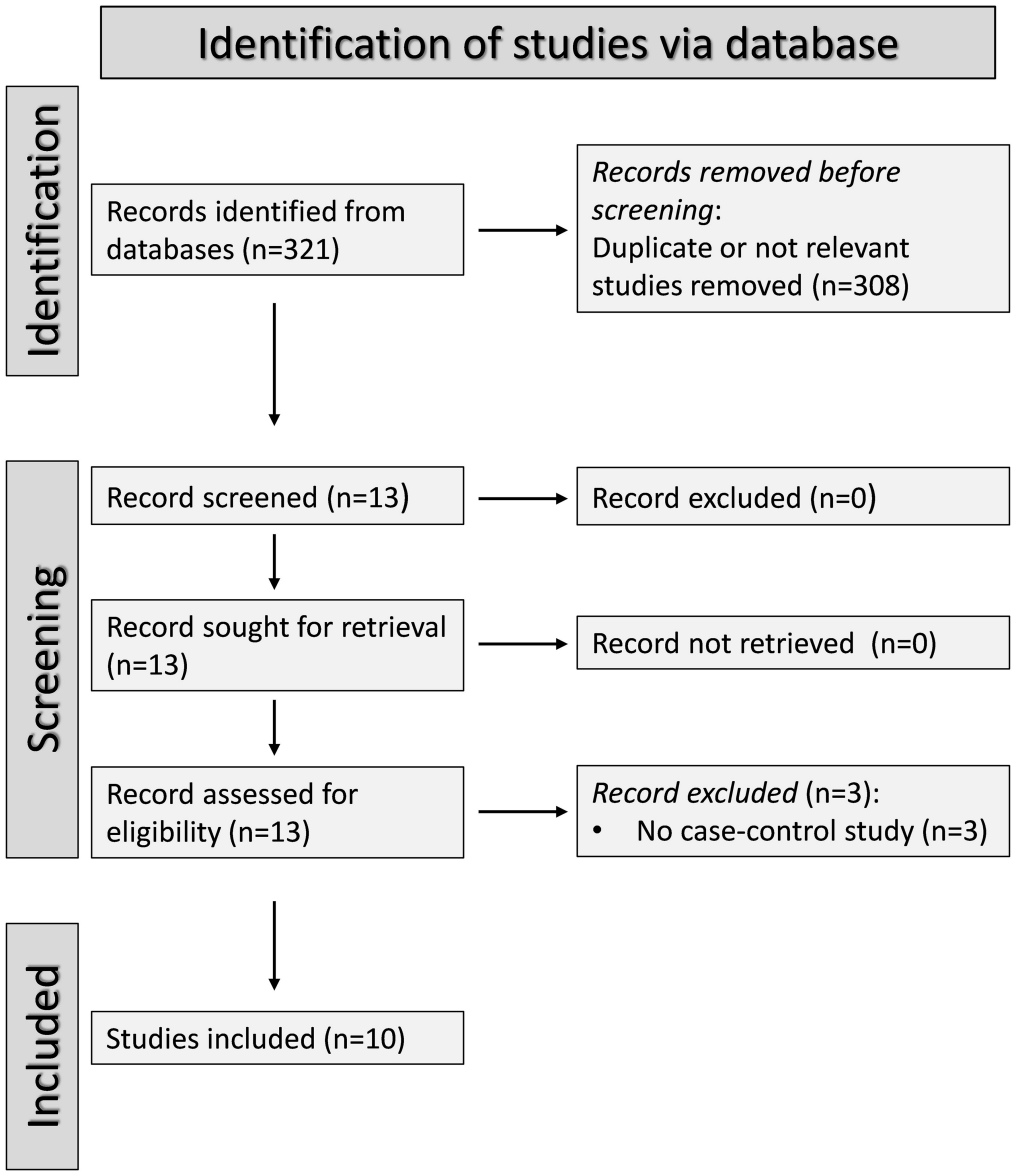
PRISMA 2020 flow diagram.

The PRISMA flow chart of the study selection is described in **Figure 1**. After initially identifying 321 articles, 308 were excluded because they were either duplicates or presented data that were not relevant to the study question. After fully revising the remaining13 articles, a further three were excluded because the study design was not case-control. Therefore, ten studies, all with a low risk of bias, were selected for analysis (46–55) (**Table 1**, **Supplementary Table 2**). SSc patients did not receive any treatment in three studies (48, 49, 51), 15% received treatment with corticosteroids in one study (47), 33.3%, 21.1%, and 13.2% received treatment with corticosteroids, penicillamine, and methotrexate, respectively, in one study (50), whereas relevant information regarding treatment was not reported in the remaining five studies (46, 52–55). The cross-sectional design of the studies identified downgraded the initial certainly of evidence to low.

**Table 1:** Characteristics of the studies investigating the NLR, PLR, and MLR values in patients with systemic sclerosis and healthy controls.

Study	Healthy controls				Patients with systemic sclerosis				Study design
	n	Age (Years)	M/F	NLR PLR MLR (Mean ± SD)	n	Age (Years)	M/F	NLR PLR MLR (Mean ± SD)	
Esheba NE et al., 2016, Egypt (46)	25	40	2/23	1.52 ± 0.54 NR NR	25	41	2/23	2.34 ± 1.04 NR NR	R
Yolbas S et al., 2016, Turkey (47)	55	45	11/44	2.43 ± 0.99 151 ± 56 NR	39	49	4/35	6.40 ± 4.43 306 ± 196 NR	P
Yang Z et al., 2017, China (49)	170	45	19/151	1.80 ± 0.76 NR 0.21 ± 0.05	33	55	4/29	2.33 ± 0.69 NR 0.28 ± 0.09	R
Jung JH et al., 2017, South Korea (48)	50	49	7/43	2.00 ± 1.07 126 ± 42 NR	88	50	10/78	3.95 ± 6.59 164 ± 101 NR	R
Kim A et al., 2020, South Korea (50)	304	55	0/304	1.32 ± 0.49 120 ± 29 NR	114	57	0/114	1.89 ± 0.95 129 ± 46 NR	P
Tezcan D et al., 2020, Turkey (51)	129	51	8/121	1.59 ± 0.36 114 ± 37 0.19 ± 0.05	129	52	7/122	2.18 ± 1.13 136 ± 72 0.29 ± 0.12	R
Yayla ME et al., 2020, Turkey (52)	50	49	9/41	1.75 ± 1.64 NR 0.16 ± 0.25	69	53	7/62	2.40 ± 5.78 NR 0.21 ± 0.59	R
Sakr BR et al., 2021, Egypt (53)	45	41	3/42	2.45 ± 1.41 NR NR	35	43	6/29	4.24 ± 2.45 NR NR	P
Li H et al., 2022, China (54)	NR	NR	NR	NR NR NR	227	53	39/188	2.59 ± 1.42 NR NR	R
Nejatifar F et al., 2023, Iran (55)	123	NR	57/66	2.10 ± 0.80 NR 0.2 ± 0.2	123	NR	14/109	2.50 ± 1.40 NR 0.2 ± 0.1	R

NR, not reported; MLR, monocyte-to-lymphocyte ratio; NLR, neutrophil-to-lymphocyte ratio; PLR, platelet-to-lymphocyte ratio.

### Neutrophil-to-lymphocyte ratio

Nine studies reported the NLR in a total of 655 SSc patients (mean age 52 years, 92% females) and 951 healthy controls (mean age 50 years, 88% females) (46–53, 55). Three studies were conducted in Turkey (47, 51, 52), two in Egypt (46, 53), two in South Korea (48, 50), one in China (49), and one in Iran (55). Six studies were retrospective (46, 48, 49, 51, 52, 55), and three prospective (47, 50, 53).

**Figure 2:**
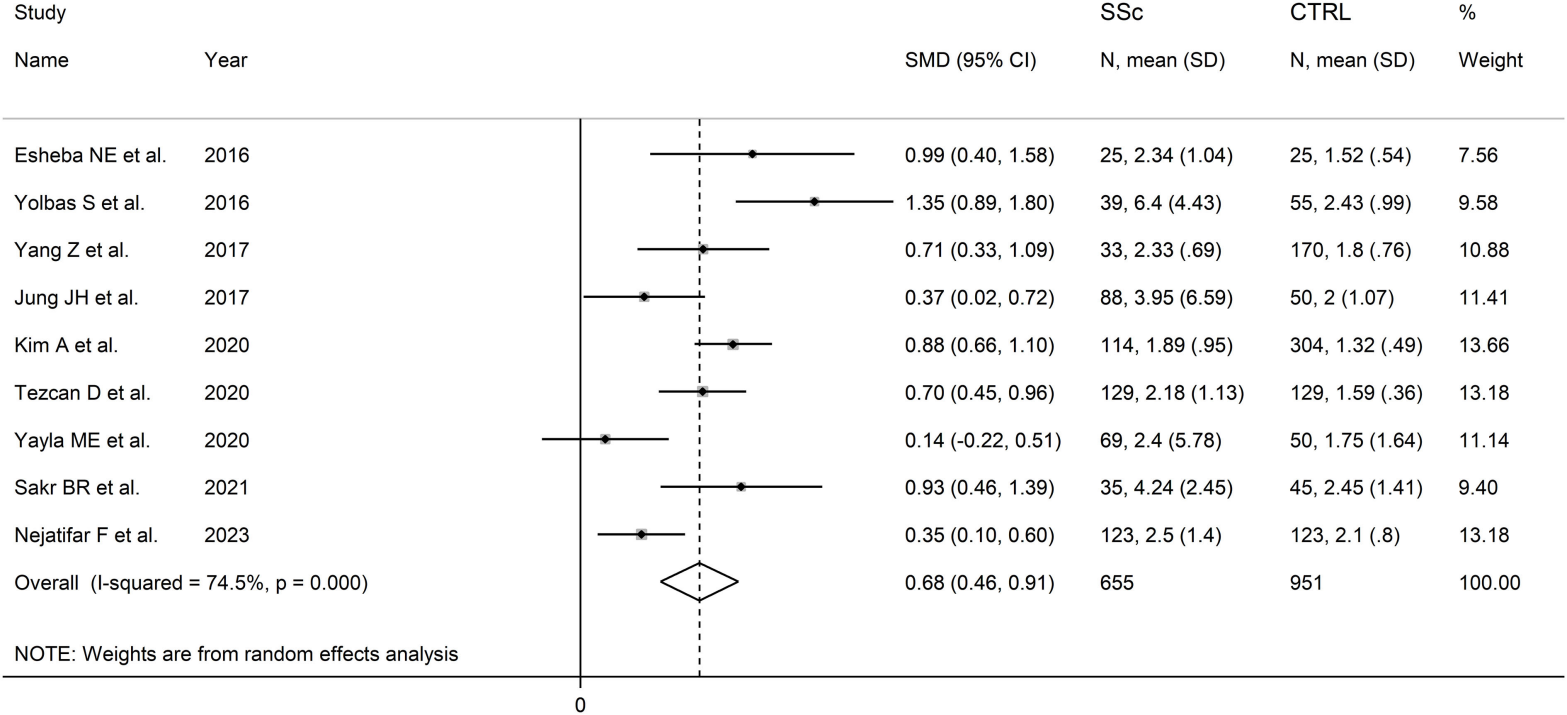
Forest plot of studies reporting the neutrophil-to-lymphocyte ratio in patients with systemic sclerosis and healthy controls.

The forest plot showed that the NLR values were significantly higher in SSc patients when compared to controls (SMD=0.68, 95% CI 0.46 to 0.91, p<0.001; I^2^ = 74.5%, p<0.001; **Figure 2**), with stable results in sensitivity analysis (corresponding pooled SMD ranging between 0.61 and 0.75; **Supplementary Figure 1**).

There was no evidence of publication bias (Begg’s test, p=0.75; Egger’s test, p=0.60). The “trim-and-fill” method identified two missing studies to be added to the left side of the funnel plot to ensure symmetry (**Supplementary Figure 2**). The resulting effect size remained significant (SMD=0.59, 95% CI 0.36 to 0.82, p<0.001).

In subgroup analysis, the pooled SMD was not significantly different (p=0.37) between studies in patients aged ≤50 years (SMD=0.89, 95% CI 0.43 to 1.35, p<0.001; I^2^ = 75.3%, p=0.007), and >50 years (SMD=0.63, 95% CI 0.34 to 0.92, p<0.001; I^2^ = 73.7%, p=0.010; **Supplementary Figure 3**). The pooled SMD was not significantly different (p=0.24) between studies with (SSc patient males/SSc patient females)/(control males/control females) ratio <1 (SMD=0.56, 95% CI 0.23 to 0.89, p=0.001; I^2^ = 81.3%, p<0.001) and (SSc patient males/SSc patient females)/(control males/control females) ratio ≥1 (SMD=0.86, 95% CI 0.69 to 1.03, p<0.001; I^2^ = 0.0%, p=0.82; **Supplementary Figure 4**), with a virtually absent between study variance in the second subgroup. Non-significant differences (p=0.65) were also observed between studies conducted in Turkey (SMD=0.72, 95% CI 0.13 to 1.30, p<0.001; I^2^ = 88.0%, p<0.001), South Korea (SMD=0.64, 95% CI 0.14 to 1.14, p=0.012; I^2^ = 82.9%, p=0.016), Egypt (SMD=0.95, 95% CI 0.59 to 1.32, p<0.001; I^2^ = 0.0%, p=0.87), and other countries (SMD=0.50, 95% CI 0.16 to 0.84, p=0.004; I^2^ = 57.6%, p=0.12; **Supplementary Figure 5**), with a virtually absent heterogeneity in the Egyptian subgroup. By contrast, we observed a significant difference (p=0.034) in pooled SMD between retrospective (SMD=0.51, 95% CI 0.29 to 0.72, p<0.001; I^2^ = 58.0%, p=0.036) and prospective studies (SMD=1.01, 95% CI 0.74 to 1.28, p<0.001; I^2^ = 39.6%, p=0.19; **Supplementary Figure 6**), with a relatively low between-study variance in both subgroups.

The overall level of certainty remained low after considering the low risk of bias in all studies, the high but partially explainable heterogeneity, the lack of indirectness, the moderate effect size (SMD=0.68) (56), and the absence of publication bias.

#### Neutrophil-to-lymphocyte ratio and interstitial lung disease

Six studies reported the NLR in a total of 326 SSc patients with ILD and 324 SSc patients without ILD (46, 48, 50–52, 54) (**Table 2**). Two studies were conducted in Turkey (51, 52), two in South Korea (48, 50), one in China (54), and one in Egypt (46). Five studies were retrospective (46, 48, 51, 52, 54) and one prospective (50).

**Table 2:** Characteristics of the studies investigating the NLR, PLR, and MLR in patients with systemic sclerosis with and without complications.

	ILD -		ILD +		PAH -		PAH +		DU -		DU +	
	n	NLR PLR MLR (Mean ± SD)	n	NLR PLR MLR (Mean ± SD)	n	NLR PLR MLR (Mean ± SD)	n	NLR PLR MLR (Mean ± SD)	n	NLR PLR MLR (Mean ± SD)	n	NLR PLR MLR (Mean ± SD)
Esheba NE et al., 2016, Egypt (46)	8	2.00 ± 0.91 NR NR	15	2.51 ± 0.98 NR NR	22	2.15 ± 0.42 NR NR	3	3.9 ± 0.56 NR NR	15	2.01 ± 0.82 NR NR	10	2.34 ± 0.99 NR NR
Yolbas S et al., 2016, Turkey (47)	NR	NR NR NR	NR	NR NR NR	NR	NR NR NR	NR	NR NR NR	NR	NR NR NR	NR	NR NR NR
Yang Z et al., 2017, China (49)	NR	NR NR NR	NR	NR NR NR	NR	NR NR NR	NR	NR NR NR	NR	NR NR NR	NR	NR NR NR
Jung JH et al., 2017, South Korea (48)	48	2.12 ± 1.71 142 ± 67 NR	40	6.13 ± 9.18 190 ± 127 NR	NR	NR NR NR	NR	NR NR NR	NR	NR NR NR	NR	NR NR NR
Kim A et al., 2020, South Korea (50)	60	1.62 ± 0.55 116 ± 35 NR	54	2.24 ± 1.67 153 ± 62 NR	NR	NR NR NR	NR	NR NR NR	79	1.71 ± 0.70 120 ± 44 NR	35	2.26 ± 1.34 162 ± 74 NR
Tezcan D et al., 2020, Turkey (51)	66	2.06 ± 0.90 126 ± 61 0.27 ± 0.09	63	2.31 ± 1.37 147 ± 83 0.32 ± 0.16	112	2.10 ± 0.47 141 ± 67 0.29 ± 0.11	17	3.38 ± 2.80 148 ± 79 0.40 ± 0.22	NR	NR NR NR	NR	NR NR NR
Yayla ME et al., 2020, Turkey (52)	21	2.40 ± 5.80 NR 0.21 ± 0.58	48	2.40 ± 5.30 NR 0.21 ± 0.37	64	2.39 ± 5.79 NR 0.08 ± 0.47	5	3.16 ± 4.77 NR 0.05 ± 0.09	54	2.31 ± 5.79 NR 0.20 ± 0.59	15	3.16 ± 1.98 NR 0.24 ± 0.32
Sakr BR et al., 2021, Egypt (53)	NR	NR NR NR	NR	NR NR NR	NR	NR NR NR	NR	NR NR NR	NR	NR NR NR	NR	NR NR NR
Li H et al., 2022, China (54)	121	2.50 ± 1.00 139 ± 59 NR	106	2.70 ± 1.90 160 ± 72 NR	NR	NR NR NR	NR	NR NR NR	NR	NR NR NR	NR	NR NR NR
Nejatifar F et al., 2023, Iran (55)	NR	NR NR NR	NR	NR NR NR	NR	NR NR NR	NR	NR NR NR	NR	NR NR NR	NR	NR NR NR

DU, digital ulcers; ILD, interstitial lung disease; NR, not reported; PAH, pulmonary artery hypertension.

**Figure 3:**
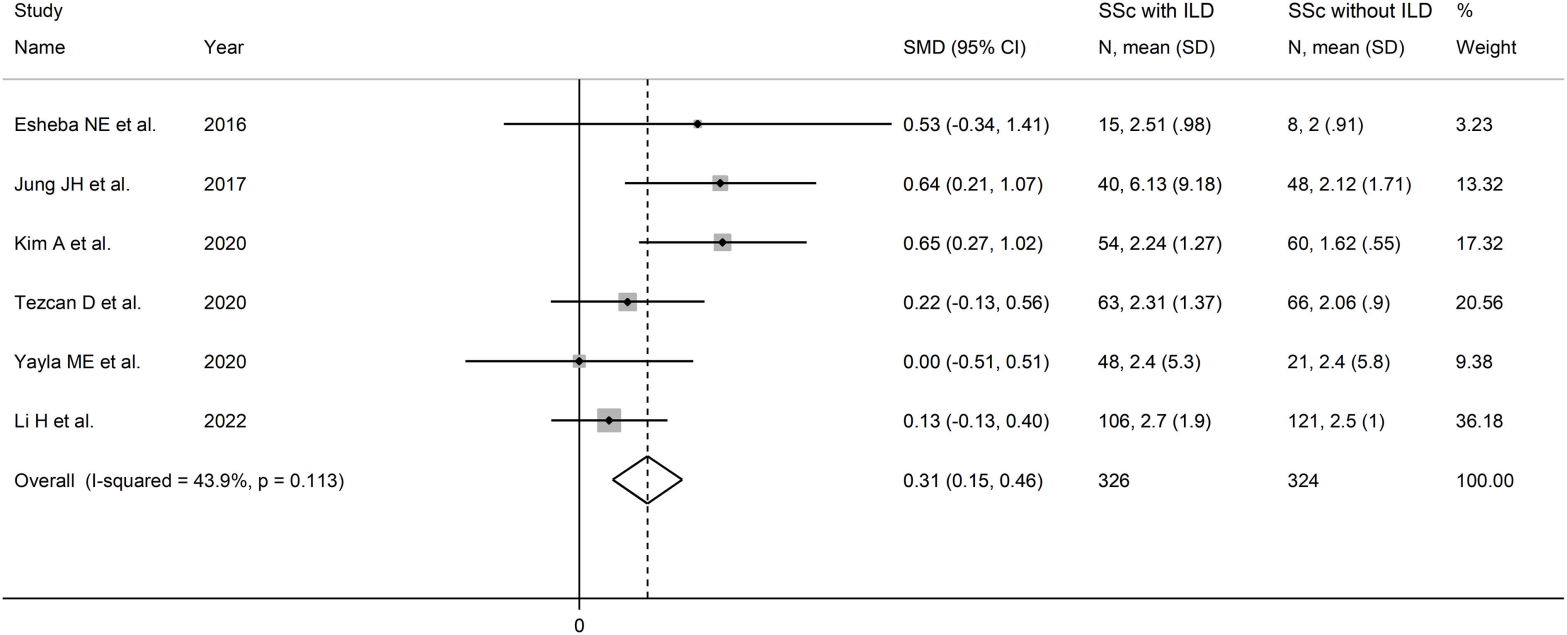
Forest plot of studies reporting the neutrophil-to-lymphocyte ratio in patients with systemic sclerosis with and without interstitial lung disease.

The forest plot showed that SSc patients with ILD had higher NLR values when compared to patients without ILD (SMD=0.31, 95% CI 0.15 to 0.46, p<0.001; I^2^ = 43.9%, p=0.11; **Figure 3**). The corresponding pooled SMD values were stable in sensitivity analysis (range between 0.25 and 0.40; **Supplementary Figure 7**).

The limited number of studies prevented the assessment of publication bias and the conduct of meta-regression analysis. In sub-group analysis, the pooled SMD was significant in studies conducted in South Korea (SMD=0.64, 95% CI 0.36 to 0.92, p<0.001; I^2^ = 0.0%, p=0.97) but not in Turkey (SMD=0.15, 95% CI -0.14 to 0.44, p=0.30; I^2^ = 0.0%, p=0.49), or other countries (SMD=0.17, 95% CI -0.08 to 0.42, p=0.19; I^2^ = 0.0%, p=0.39; **Supplementary Figure 8**), with a virtually absent heterogeneity in all subgroups.

The overall level of certainty was downgraded to very low because of the lack of assessment of publication bias.

#### Neutrophil-to-lymphocyte ratio and pulmonary arterial hypertension

Three studies, all retrospective, investigated the NLR in a total of 198 SSc patients without PAH and 25 SSc patients with PAH (46, 51, 52) (**Table 2**). Two studies were conducted in Turkey (51, 52), and one in Egypt (46).

**Figure 4:**
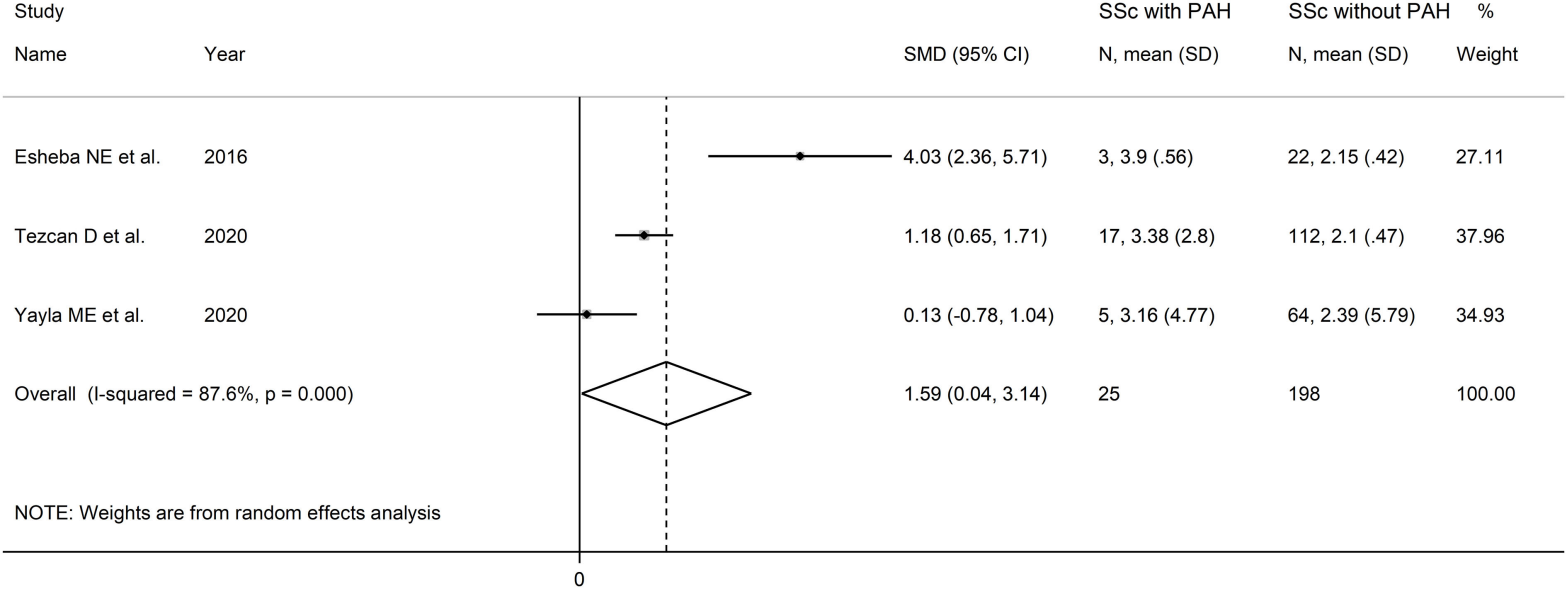
Forest plot of studies examining the neutrophil-to-lymphocyte ratio in patients with systemic sclerosis with and without pulmonary arterial hypertension.

The forest plot showed that SSc patients with PAH had higher NLR values when compared to SSc patients without PAH (SMD=1.59, 95% CI 0.04 to 3.1, p=0.045; I^2^ = 87.6%, p<0.001; **Figure 4**). Assessment of sensitivity, publication bias, meta-regression and sub-group analyses could not be performed because of the small number of studies.

The overall level of certainty was downgraded to very low after considering the low risk of bias in all studies, the high and unexplainable heterogeneity (downgrade one level), the lack of indirectness, the large effect size (SMD=1.59, upgrade one level) (56), and the lack of assessment of publication bias (downgrade one level).

#### Neutrophil-to-lymphocyte ratio and digital ulcers

Three studies reported the NLR in a total of 148 SSc patients without DU and 60 SSc patients with DU (46, 50, 52) (**Table 2**). One study was conducted in Egypt (46), one in South Korea (50), and one in Turkey (52). Two studies were retrospective (46, 52), and one prospective (50).

**Figure 5:**
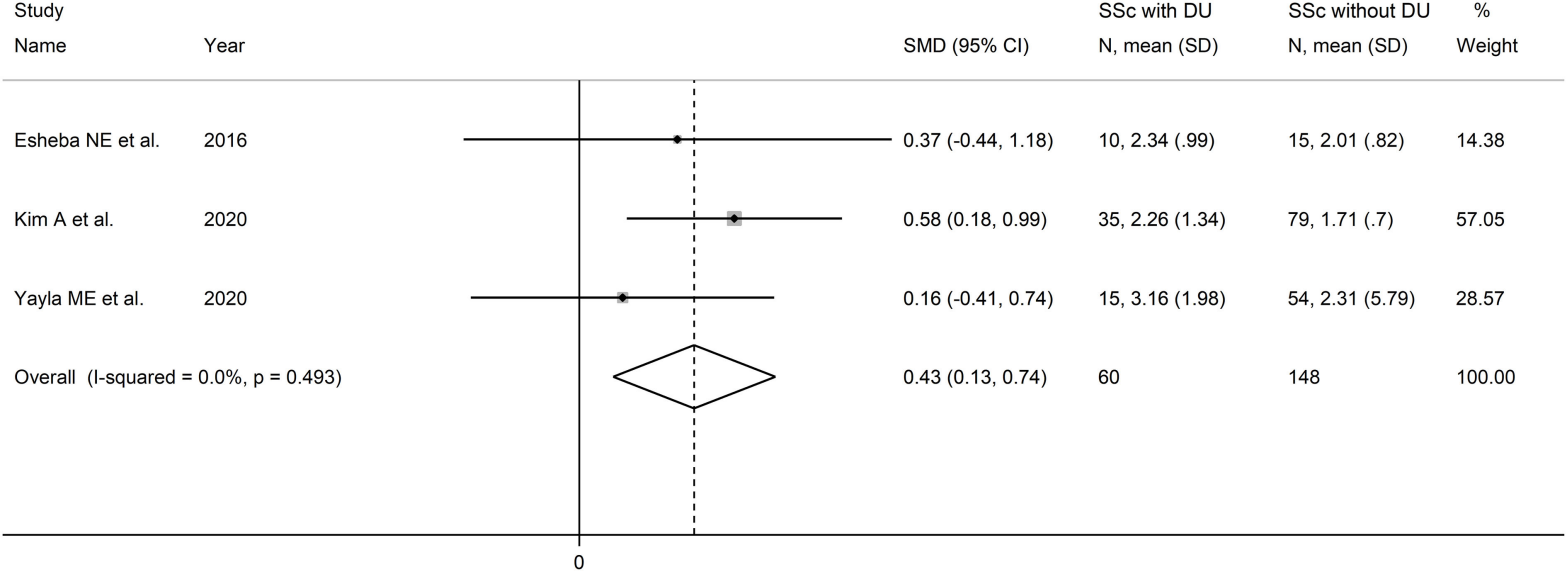
Forest plot of studies examining the neutrophil-to-lymphocyte ratio in patients with systemic sclerosis with and without digital ulcers.

The forest plot showed that the NLR values were significantly higher in SSc patients with DU when compared to SSc patients without DU (SMD=0.43, 95% CI 0.13 to 0.74, p=0.006; I^2^ = 0.0%, p=0.49; **Figure 5**). Assessment of sensitivity, publication bias, and meta-regression, and sub-group analyses could not be performed because of the small number of studies.

The overall level of certainty was downgraded to very low after considering the low risk of bias in all studies, the absent heterogeneity, the lack of indirectness, the moderate effect size (SMD=0.43) (56), and the lack of assessment of publication bias (downgrade one level).

### Platelet-to-lymphocyte ratio

Four studies reported PLR values in a total of 370 SSc patients (mean age 53 years, 94% females) and 578 healthy controls (mean age 52 years, 95% females) (47, 48, 50, 51) (**Table 2**). Two studies were conducted in Turkey (47, 51), and two in South Korea (48, 50). Two studies were retrospective (48, 51), and the remaining two prospective (47, 50).

**Figure 6:**
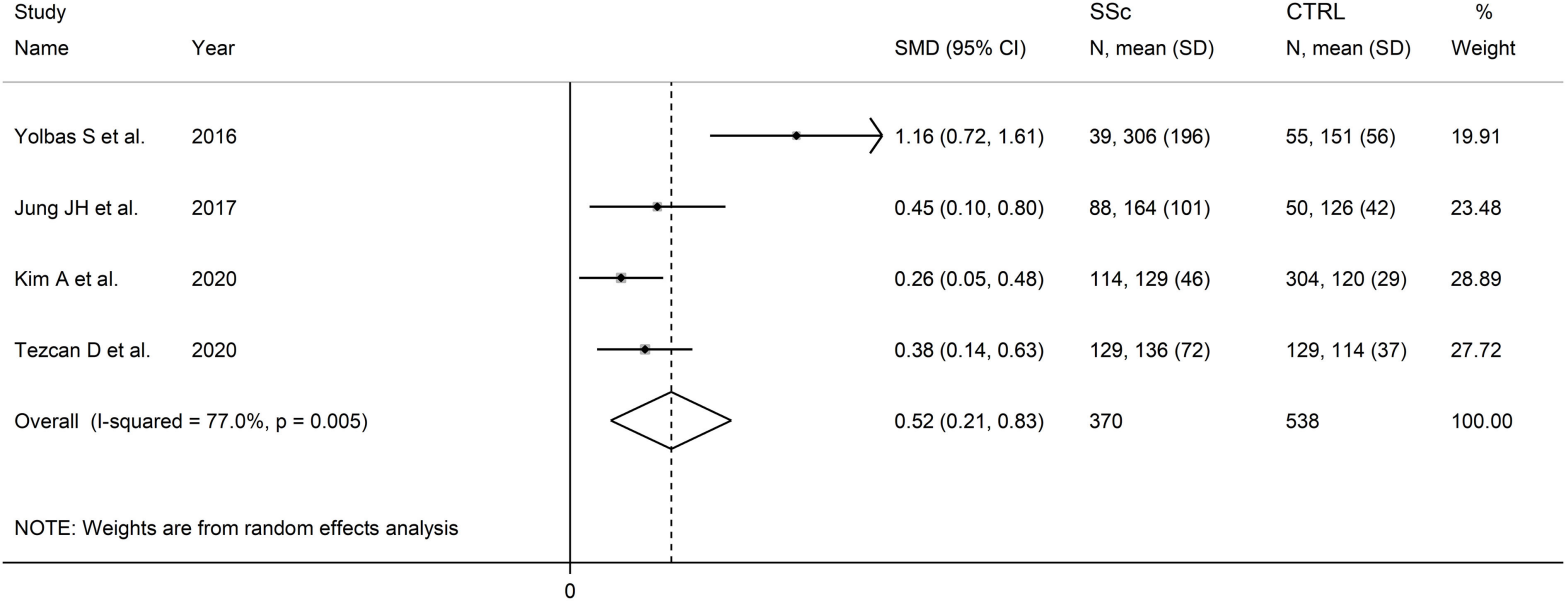
Forest plot of studies examining the platelet-to-lymphocyte ratio in patients with systemic sclerosis and healthy controls.

The forest plot showed the PLR values were significantly higher in SSc patients when compared to controls (SMD=0.52, 95% CI 0.21 to 0.83, p=0.001; I^2^ = 77.0%, p=0.005; **Figure 6**). Assessment of sensitivity, publication bias, meta-regression, sub-group analyses could not be performed because of the small number of studies.

The overall level of certainty was downgraded to very low after considering the high and unexplained heterogeneity and the lack of assessment of publication bias.

#### Platelet-to-lymphocyte ratio and interstitial lung disease

Four studies investigated the PLR in a total of 263 SSc patients with ILD and 295 SSc patients without ILD (48, 50, 51, 54) (**Table 2**). Two studies were conducted in South Korea (48, 50), one in Turkey (51), and one in China (54). Three studies were retrospective (48, 51, 54), and one prospective (50).

**Figure 7:**
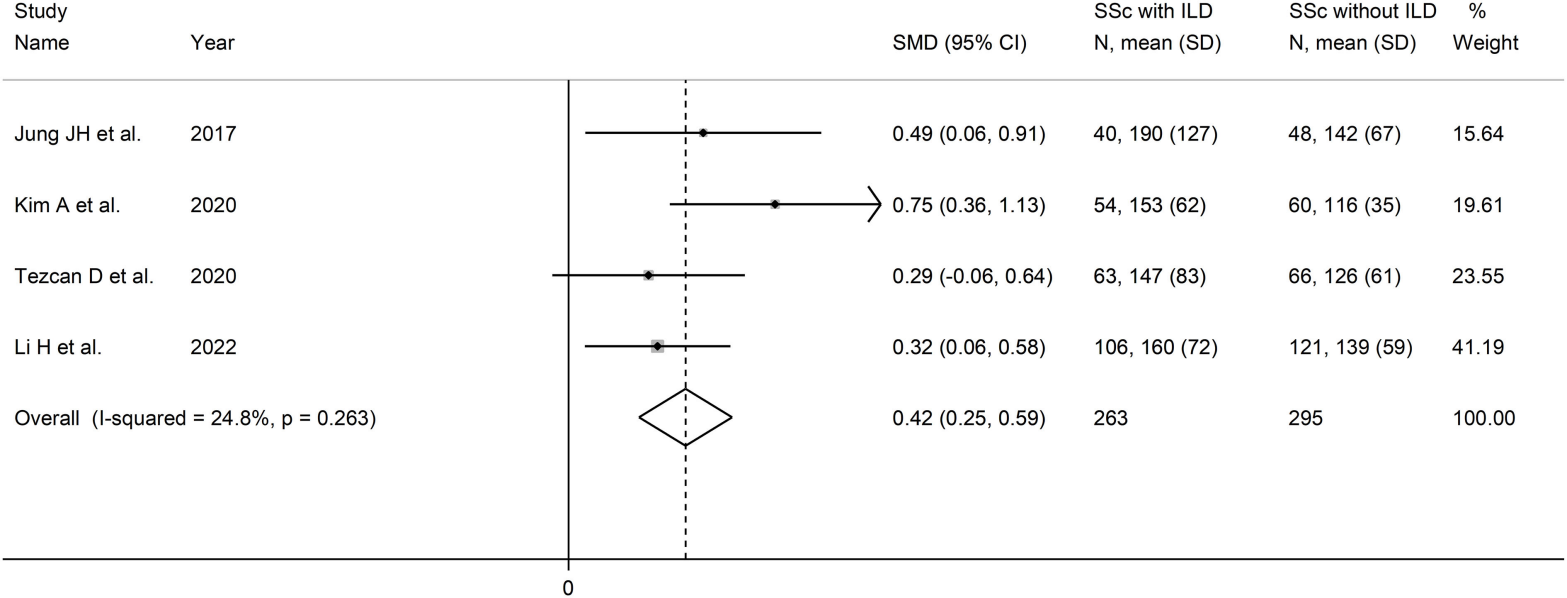
Forest plot of studies examining the platelet-to-lymphocyte ratio in patients with systemic sclerosis with and without interstitial lung disease.

The forest plot showed that the PLR values were significantly higher in SSc patients with ILD when compared to SSc patients without ILD (SMD=0.42, 95% CI 0.25 to 0.59, p<0.001; I^2^ = 24.8%, p=0.26; **Figure 7**). Assessment of sensitivity, publication bias, meta-regression, and subgroup analysis could not be performed because of the small number of studies.

The overall level of certainty was downgraded to very low after considering the lack of assessment of publication bias.

#### Platelet-to-lymphocyte ratio and pulmonary arterial hypertension

One study reported that SSc patients with PAH had significantly higher PLR values when compared to SSc patients without PAH (median 147.0 (IQR:111.6) vs. 125.6 (IQR: 82.3), p<0.001) (51).

#### Platelet-to-lymphocyte ratio and digital ulcers

One study reported that SSc patients with DU had significantly higher PLR values when compared to SSc patients without DU (median 148.07 (IQR, 121.33 to 217.66) vs. 115.67 (IQR, 93.5 to 151.88), p=0.001) (50).

### Monocyte-to-lymphocyte ratio

Four studies, all retrospective, reported the MLR in a total of 354 SSc patients (mean age 53 years, 90% females) and 472 healthy controls (mean age 48 years, 77% females) (49, 51, 52, 55). Two studies were performed in Turkey (51, 52), one in China (49), and one in Iran (55).

**Figure 8:**
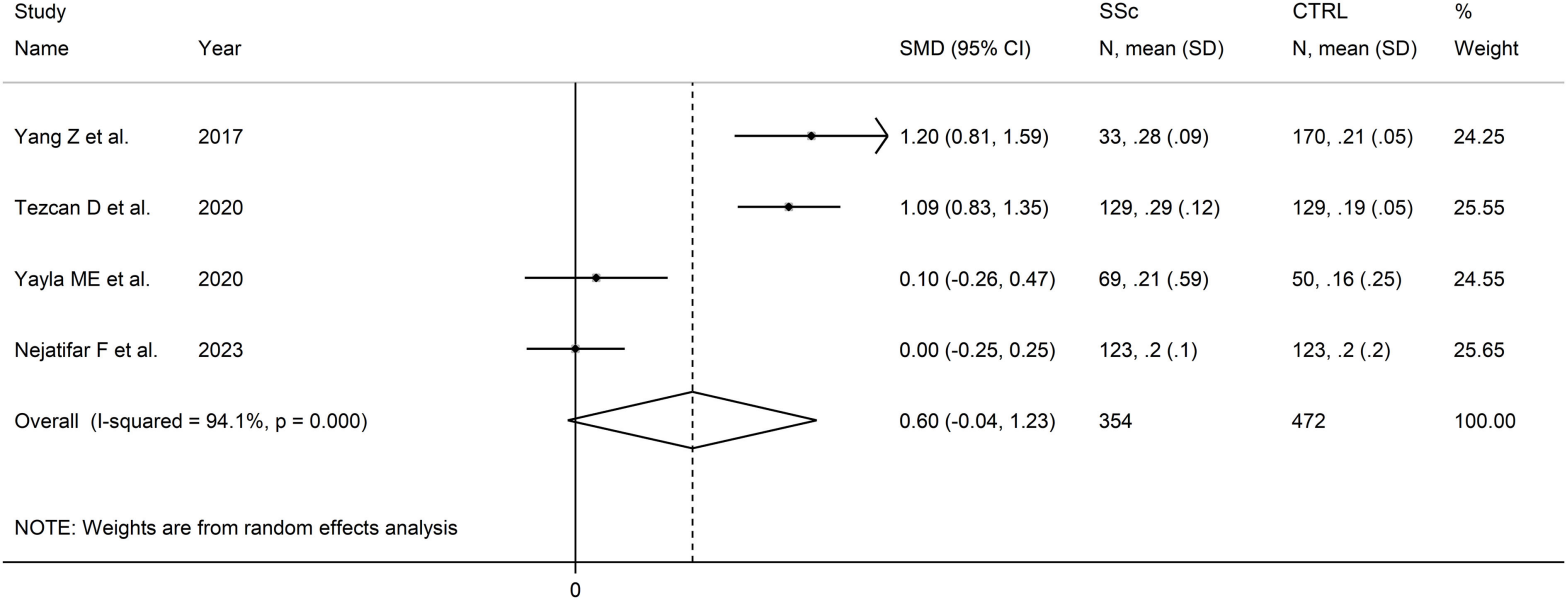
Forest plot of studies examining the monocyte-to-lymphocyte ratio in patients with systemic sclerosis and healthy controls.

The forest plot showed that that MLR values were non-significantly higher in SSc patients when compared to controls (SMD=0.60, 95% CI -0.04 to 1.23, p=0.066; I^2^ = 94.1%, p<0.001; **Figure 8**). Assessment of sensitivity, publication bias, meta-regression, and subgroup analyses could not be performed because of the small number of studies.

The overall level of certainty was downgraded to very low after considering the high and unexplained heterogeneity and the lack of assessment of publication bias.

#### Monocyte-to-lymphocyte ratio and interstitial lung disease

Two studies reported the MLR in a total of 111 SSc patients with ILD and 87 SSc patients without ILD (51, 52) (**Table 2**). Both studies were conducted in Turkey and were retrospective.

**Figure 9:**
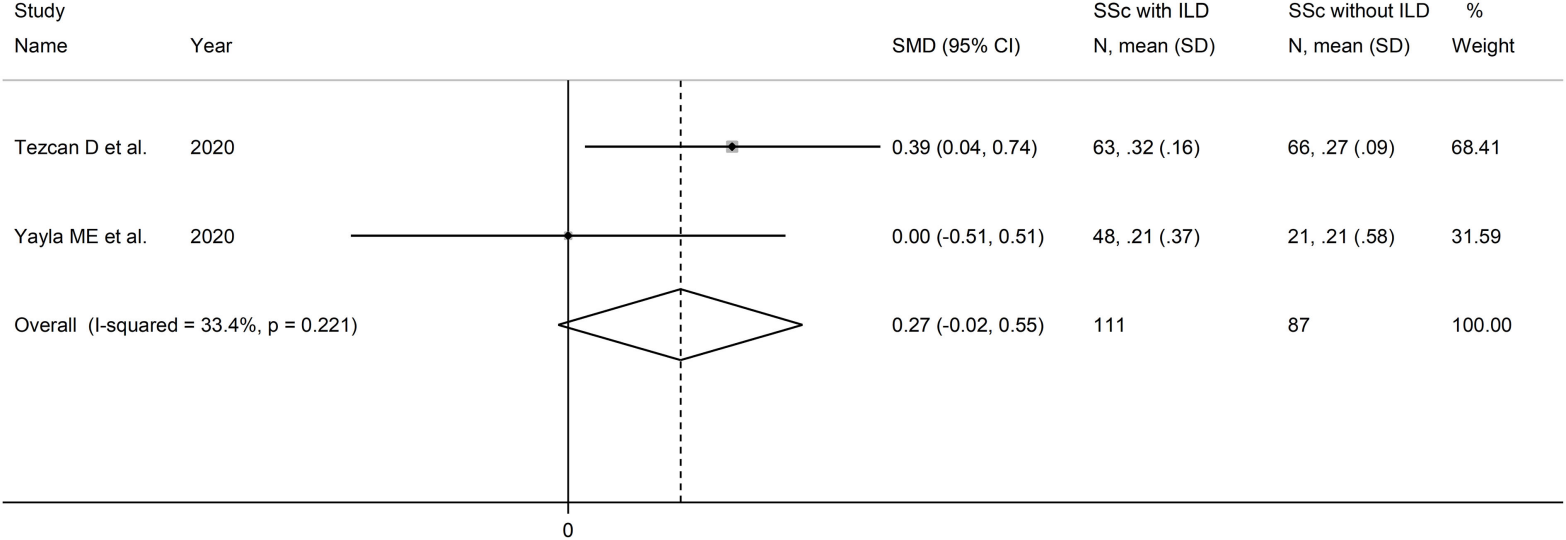
Forest plot of studies examining the monocyte-to-lymphocyte ratio in patients with systemic sclerosis with and without interstitial lung disease.

The forest plot showed that MLR values were non-significantly higher in SSc patients with ILD when compared to SSc patients without ILD (SMD=0.27, 95% CI -0.02 to 0.55, p=0.071; I^2^ = 33.4%, p=0.22; **Figure 9**). Assessment of sensitivity, publication bias, meta-regression, and subgroup analyses could not be performed because of the small number of studies.

The overall level of certainty was downgraded to very low after considering the lack of assessment of publication bias.

#### Monocyte-to-lymphocyte ratio and pulmonary arterial hypertension

Two studies reported the MLR in a total of 176 SSc patients without PAH and 22 patients with PAH (51, 52) (**Table 2**). Both studies were conducted in Turkey and were retrospective.

**Figure 10:**
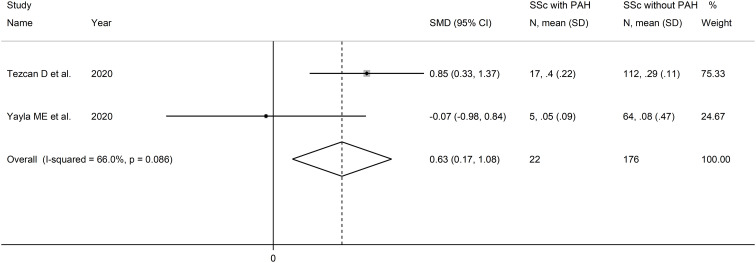
Forest plot of studies examining the monocyte-to-lymphocyte ratio in patients with systemic sclerosis with and without pulmonary arterial hypertension.

The forest plot showed that PLR values were significantly higher in SSc patients with PAH when compared to SSc patients without PAH (SMD=0.63, 95% CI 0.17 to 1.08, p=0.007; I^2^ = 66.0%, p=0.086; **Figure 10**). Assessment of sensitivity, publication bias, meta-regression and sub-group analyses could not be performed because of the small number of studies.

The overall level of certainty was downgraded to very low after considering the lack of assessment of publication bias.

#### Monocyte-to-lymphocyte ratio and digital ulcers

One study reported that SSc patients with DU had significantly higher MLR values when compared to SSc patients without DU (median 0.24 (IQR:0.43) vs. 0.20 (IQR: 0.79), p=0.007) (52).

## Discussion

The results of this systematic review and meta-analysis have shown that a) the NLR and the PLR are significantly higher in SSc patients vs. healthy controls, and b) the NLR, PLR, and MLR are significantly higher in SSc patients with specific complications (ILD, PAH, and DU for the NLR; ILD for the PLR; PAH for the MLR) vs. SSc patients without complications. Non-significant trends towards higher MLR values have also been observed in SSc patients vs. controls and in SSc patients with ILD vs. those without. Subgroup analysis for studies investigating the NLR in SSc patients and controls showed similar effect sizes regardless of age, male to female ratio, and study country. However, there was a significant difference in effect size between retrospective and prospective studies. Subgroup analysis for studies investigating the NLR in SSc patients with vs. without ILD showed differences in the significance of the effect size according to the study country. Subgroup analyses for other hematological indices and specific complications could not be conducted because of the limited number of studies. Taken together, these results suggest that specific blood cell-derived indices of inflammation, particularly the NLR and PLR, may be useful in assisting clinicians to diagnose SSc, the presence of specific complications, e.g., ILD, PAH, and DU, and potentially, to monitor the temporal progression of the disease and the response to pharmacological treatments. The negligible costs associated with the determination of the NLR, PLR, and MLR make their routine use particularly attractive in a patient group that faces significant challenges, including the lack of robust measures of SSc activity and the often fluctuating natural history of the disease (57, 58).

Recent studies have reported that the neutrophil count plays an important pathophysiological role in SSc. For example, in a study of 447 SSc patients, a higher baseline neutrophil count was significantly associated with diffuse skin disease (p<0.001), a higher baseline modified Rodnan skin thickness score (p<0.001), and a lower forced vital capacity (FVC%, p=0.03). Furthermore, a relative neutrophilia predicted lower FVC% during follow-up (point estimate -4.74, 95% CI -8.29 to -1.20, p=0.009), whereas higher lymphocyte counts were significantly associated with higher FVC% over time (point estimate 1.43, 95% CI 0.45 to 2.40, p=0.004). Notably, a higher neutrophil count also independently predicted a higher mortality (p=0.002) whereas a higher lymphocyte count independently predicted a lower mortality (p=0.001), after adjusting for age, sex, and race (18). Platelets also exhibit a state of persistent activation in SSc. Such state is likely to be triggered by chronic endothelial dysfunction and vascular damage and the activation of the innate and adaptative immune systems in these patients (19–21). Therefore, an increase in platelet count is commonly observed in SSc patients, particularly in those with a concomitant state of chronic inflammation (22). Experimental evidence also suggests an important role of monocytes in the pathophysiology of SSc (23, 27). Studies have shown that CD16-positive, non-classical, monocyte count, including monocytes expressing the type II interferon inducible marker, CXCL10, was higher in SSc patients when compared to healthy controls and was associated with an increased risk of fibrotic manifestations., e.g., ILD and a higher modified Rodnan skin score (24–26). Pending further research to identify the molecular and cellular mechanisms underpinning the detrimental effects of high neutrophil, platelet, and monocyte counts, and the potential protective effects of high lymphocyte counts, the assessment of the NLR, PLR, and MLR may optimally capture the relative alterations of these blood cell types in the assessment of patients with SSc, including the presence of specific complications. However, larger, accurately designed prospective studies are warranted to confirm our findings and to accurately determine the diagnostic performance of the NLR, PLR, and MLR, singly or in combination with clinical parameters and/or other available biomarkers, to justify their routine use in clinical practice.

One important observation in our subgroup analyses of studies investigating the NLR in SSc patients and healthy controls was that the effect size of the between-group difference was similar in studies conducted in different countries. However, it is important to emphasize that our systematic search captured studies from a limited number of countries located in Asia and Africa. Therefore, our results need confirmation in other patient populations, specifically from Europe and North and South America. This is particularly important as epidemiological studies have reported the presence of significant ethnic-based differences in the NLR in subjects without autoimmune disorders. For example, a study analyzing data from the National Health and Nutrition Examination Survey in USA reported that African American and Hispanic participants had significantly lower NLR values when compared to non-Hispanic white participants (59). Similar trends have been observed in other studies (60, 61).

Our systematic review and meta-analysis has a number of strengths, including the comprehensive assessment of three blood cell-derived inflammatory indices in patients with SSc with and without specific complications, the assessment of possible associations between the effect size and pre-defined study and patient characteristics, and a robust evaluation of the risk of bias and the certainty of evidence. Important limitations include the relatively small number of studies identified, which precluded the conduct of meta-regression and subgroup analyses for the PLR and MLR, the lack of studies investigating other important complications in patients with SSc, e.g., renal crisis, gastrointestinal and musculoskeletal involvement (2), and, as previously discussed, the lack of relevant evidence in SSc patients from specific geographical locations, particularly Europe and North and South America. A further limitation is the lack of information provided on the pharmacological treatment for SSc in five studies (46, 52–55), with three additional studies investigating treatment naïve SSc patients (48, 49, 51). This prevented the assessment of the possible effect of immunosuppressive therapies on the NLR, PLR, and MLR values, a phenomenon reported in other studies in patients with and without autoimmune disorders (62, 63).

In conclusion, our systematic review and meta-analysis has shown the potential utility of blood cell-derived indices of inflammation, particularly the NLR and PLR, in the diagnosis of SSc and associated complications, monitoring of disease activity, and assessment of the effect of treatments. However, additional studies are required to confirm these observations in different ethnic groups and determine whether the NLR and PLR can enhance the diagnostic performance of clinical assessment and existing biomarkers in this patient group in routine practice.

## Data availability statement

The original contributions presented in the study are included in the article/**Supplementary Material**. Further inquiries can be directed to the corresponding author.

## Author contributions

AZ: Conceptualization, Data curation, Formal analysis, Methodology, Validation, Writing – review & editing. AM: Methodology, Supervision, Validation, Writing – original draft, Writing – review & editing.
