# Supplementary material for: The association between the neutrophil-to-lymphocyte ratio, platelet-to-lymphocyte ratio, and monocyte-to-lymphocyte ratio and systemic sclerosis and its complications: a systematic review and meta-analysis
Source: Front Immunol. 2024 May 10;15:1395993. doi: 10.3389/fimmu.2024.1395993 (PMC11116674; doi:10.3389/fimmu.2024.1395993)
Supplement: Supplementary file 1 [file DataSheet_1.docx]

**Supplementary figure legends**

**Supplementary Figure 1.** Sensitivity analysis of the association between the neutrophil-to-lymphocyte ratio and systemic sclerosis.

**Supplementary Figure 2.** Funnel plot of studies investigating the association between the neutrophil-to-lymphocyte ratio and systemic sclerosis after “trimming-and-filling”. Dummy studies and genuine studies are represented by enclosed circles and free circles, respectively.

**Supplementary Figure 3.** Forest plot of studies examining NLR of controls and patients according to patient age.

**Supplementary Figure 4.** Forest plot of studies reporting the neutrophil-to-lymphocyte ratio in patients with systemic sclerosis and healthy controls according to the male to female ratio in patients and controls.

**Supplementary Figure 5.** Forest plot of studies reporting the neutrophil-to-lymphocyte ratio in patients with systemic sclerosis and healthy controls according to the country where the study was conducted.

**Supplementary Figure 6.** Forest plot of studies reporting the neutrophil-to-lymphocyte ratio in patients with systemic sclerosis and healthy controls according to study design.

**Supplementary Figure 7.** Sensitivity analysis of the association between the neutrophil-to-lymphocyte ratio and interstitial lung disease in patients with systemic sclerosis.

**Supplementary Figure 8.** Forest plot of studies examining the neutrophil-to-lymphocyte ratio in patients with systemic sclerosis with and without interstitial lung disease according to the country where the study has been conducted.
