# Supplementary figures and images for: The association between the neutrophil-to-lymphocyte ratio, platelet-to-lymphocyte ratio, and monocyte-to-lymphocyte ratio and systemic sclerosis and its complications: a systematic review and meta-analysis
Source: Front Immunol. 2024 May 10;15:1395993. doi: 10.3389/fimmu.2024.1395993 (PMC11116674; doi:10.3389/fimmu.2024.1395993)

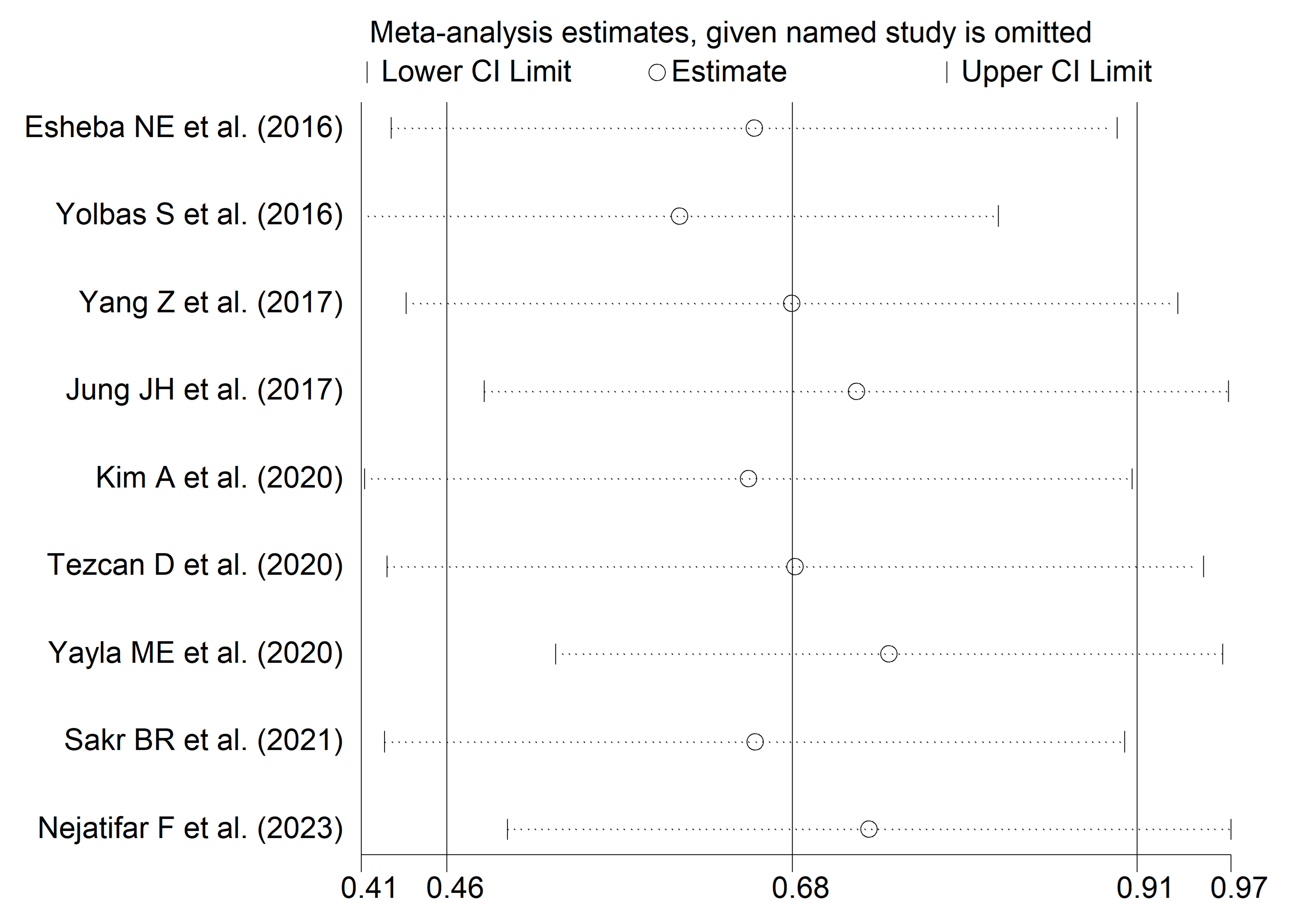

Supplement: Supplementary file 2 [file Image_1.tif]

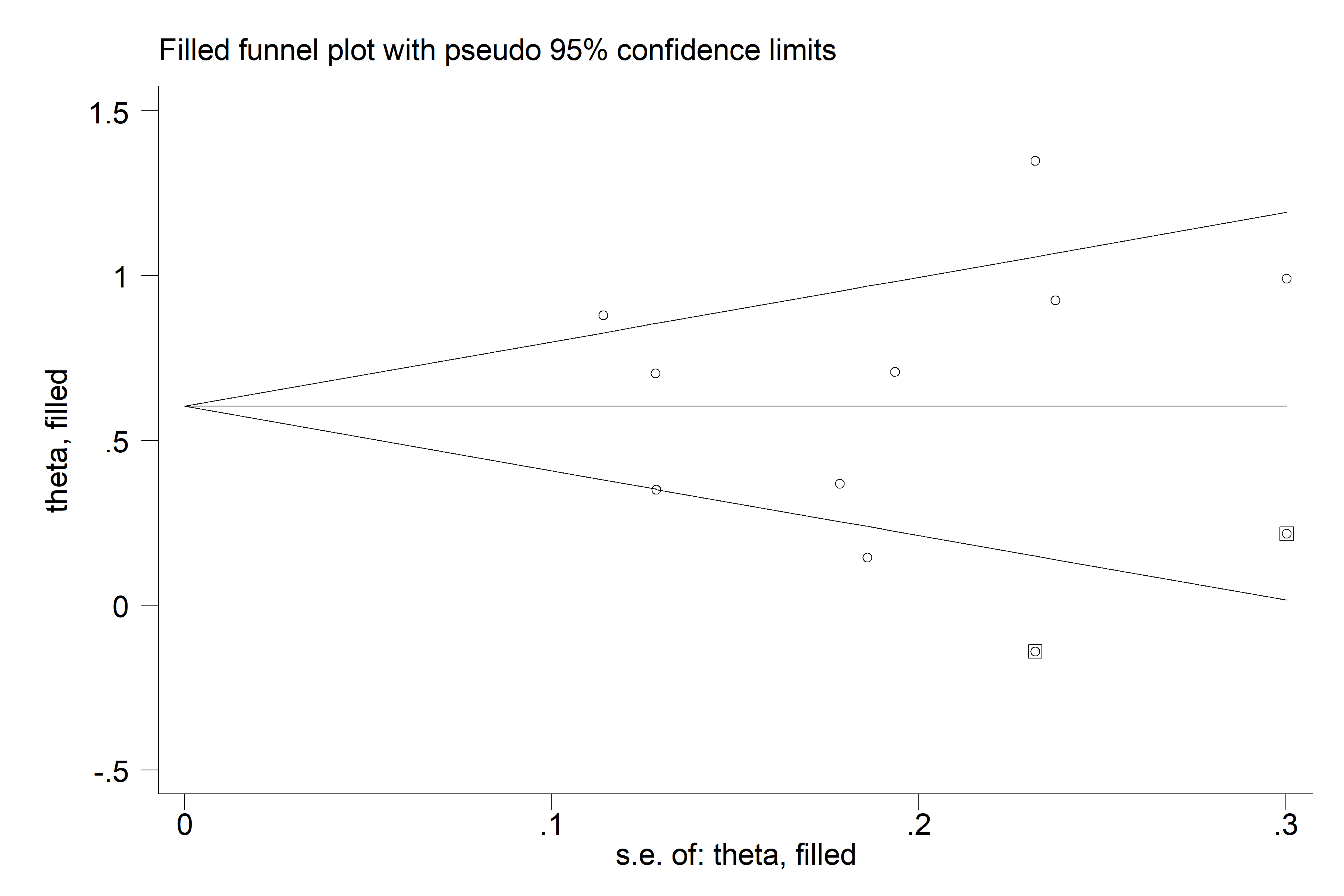

Supplement: Supplementary file 3 [file Image_2.tif]

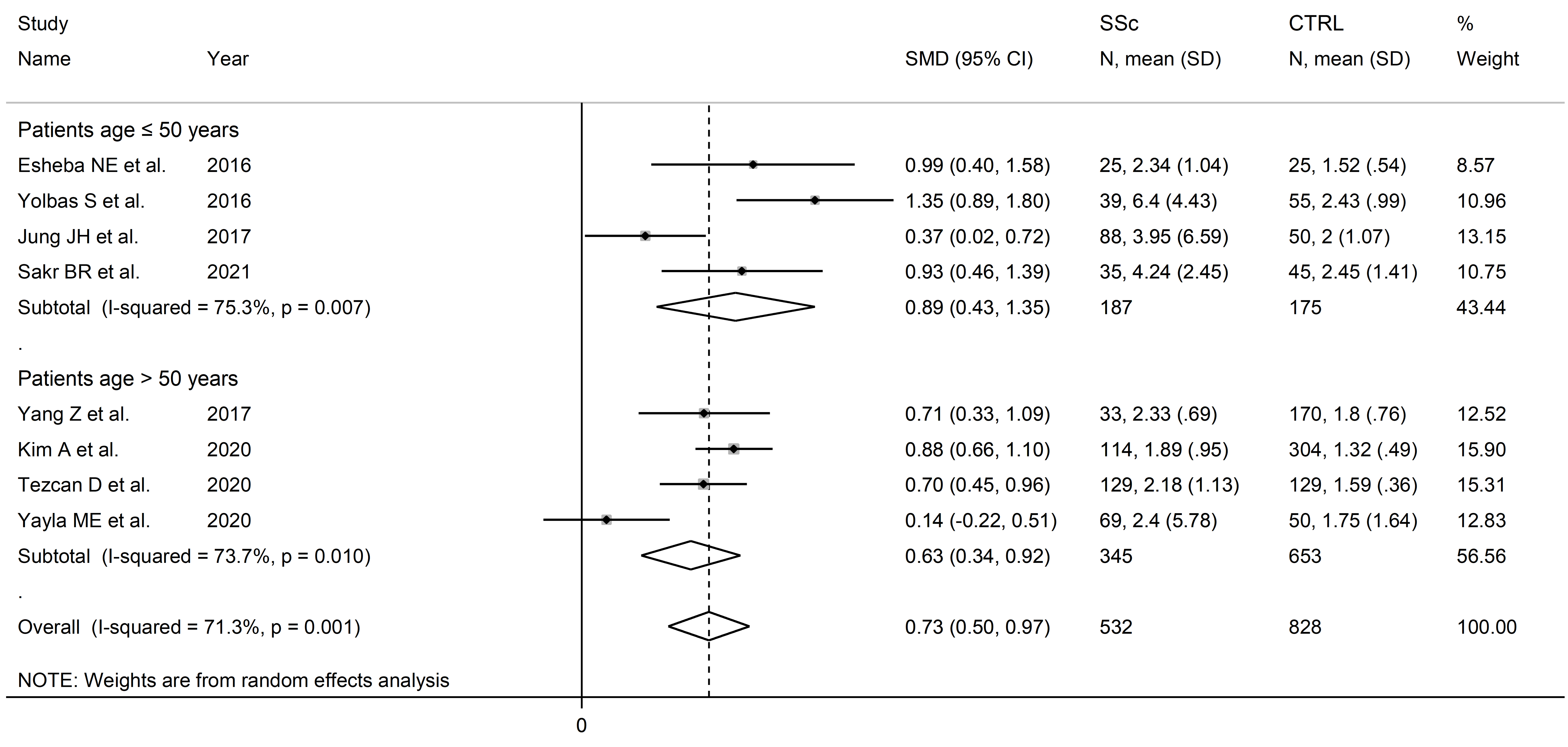

Supplement: Supplementary file 4 [file Image_3.tif]

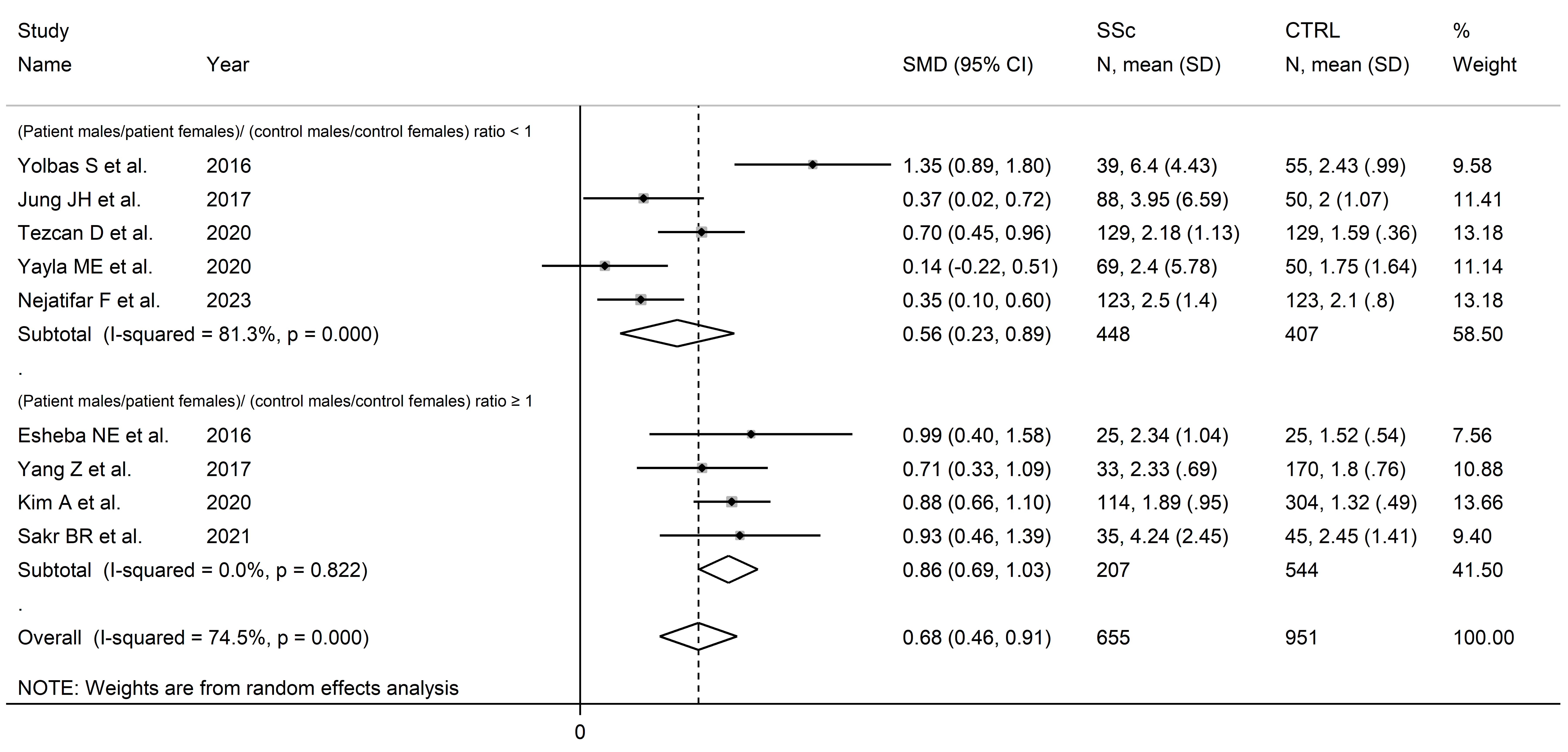

Supplement: Supplementary file 5 [file Image_4.tif]

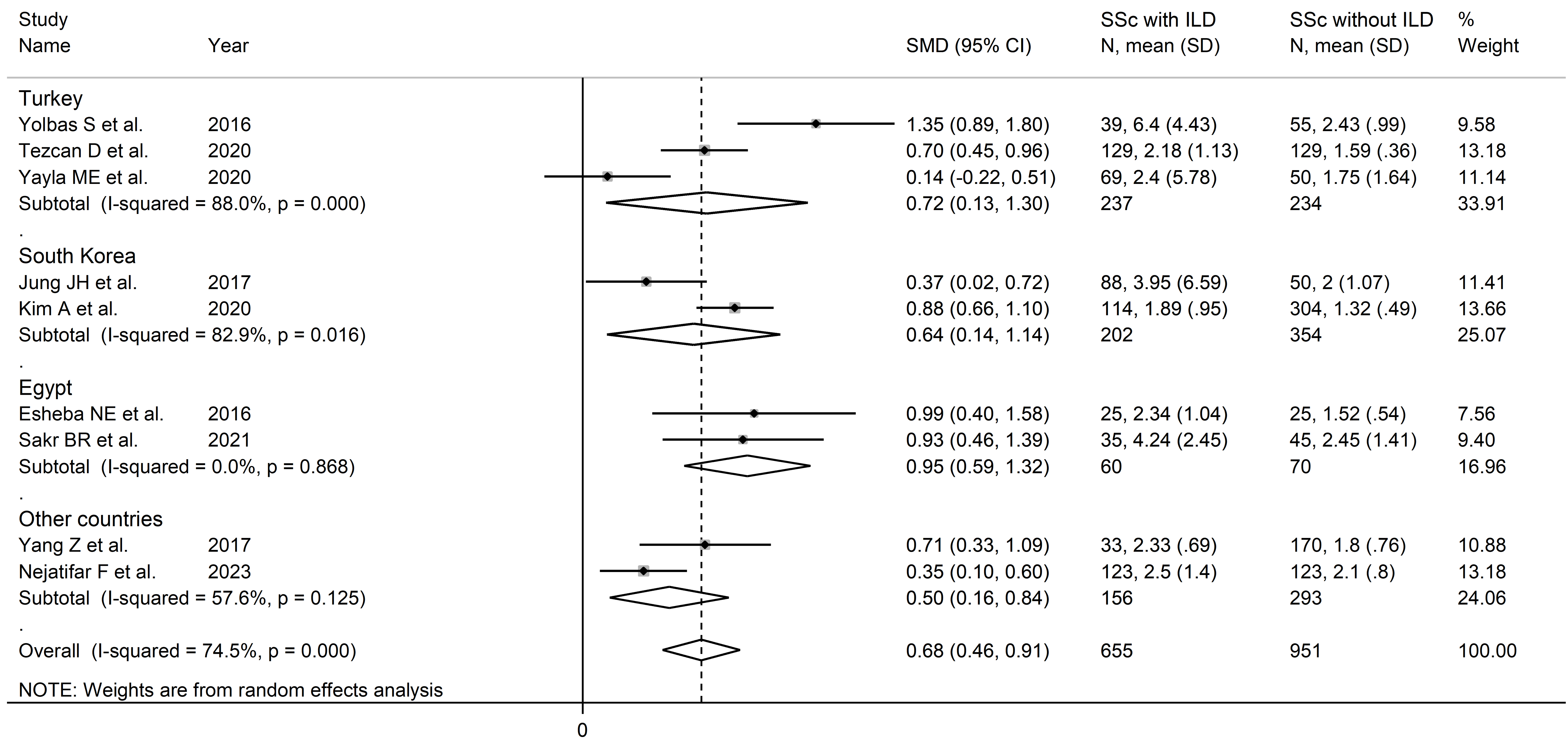

Supplement: Supplementary file 6 [file Image_5.tif]

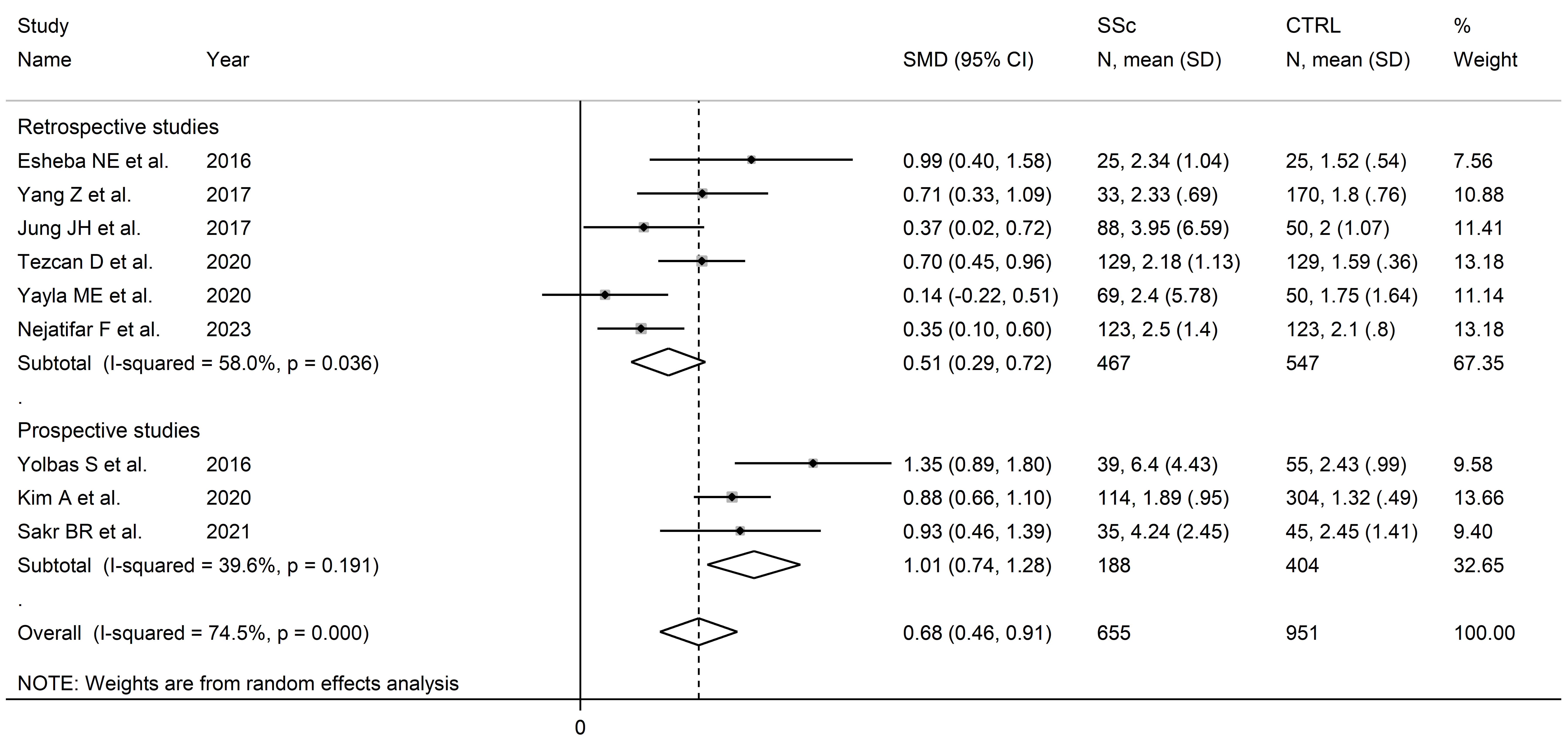

Supplement: Supplementary file 7 [file Image_6.tif]

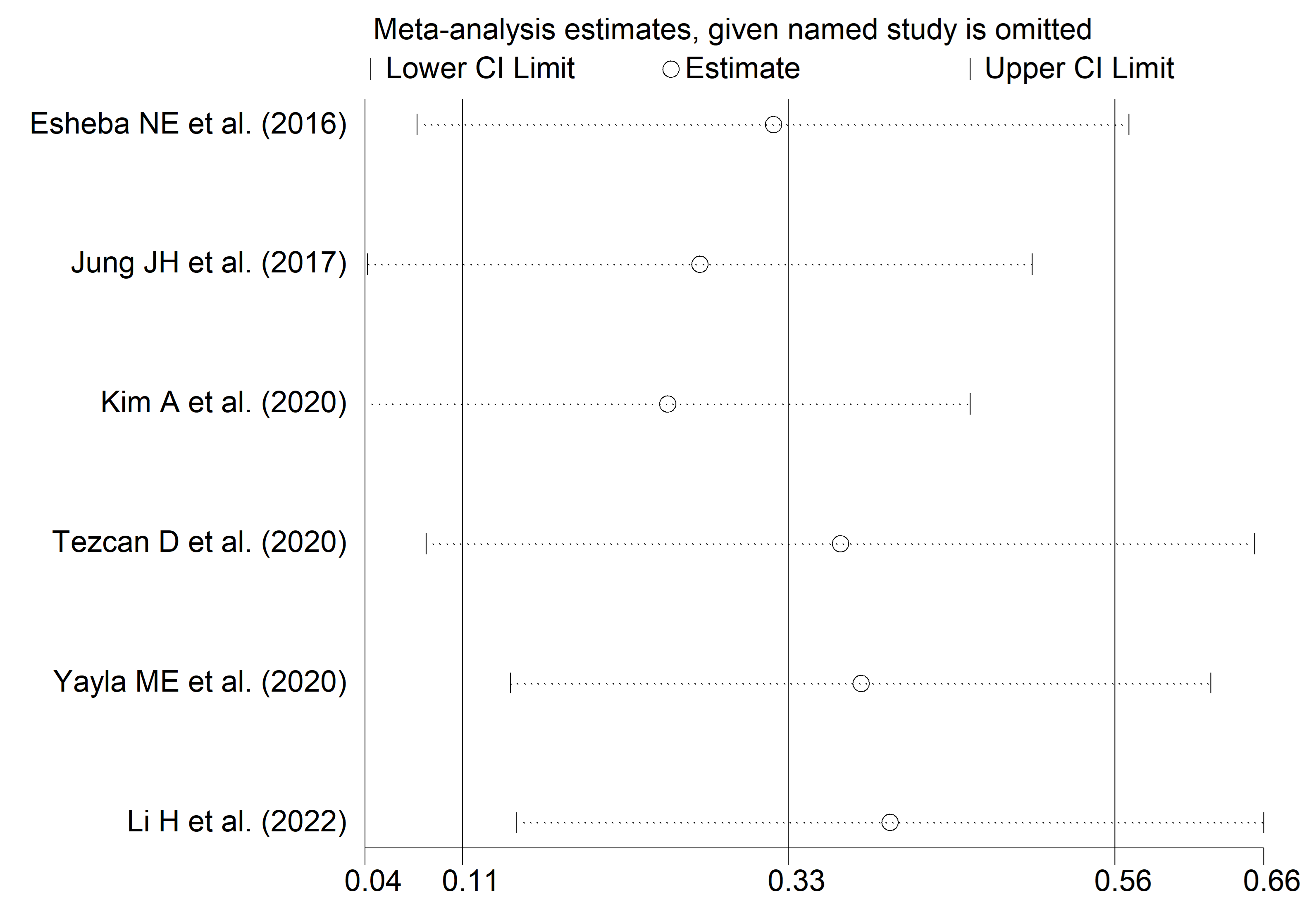

Supplement: Supplementary file 8 [file Image_7.tif]

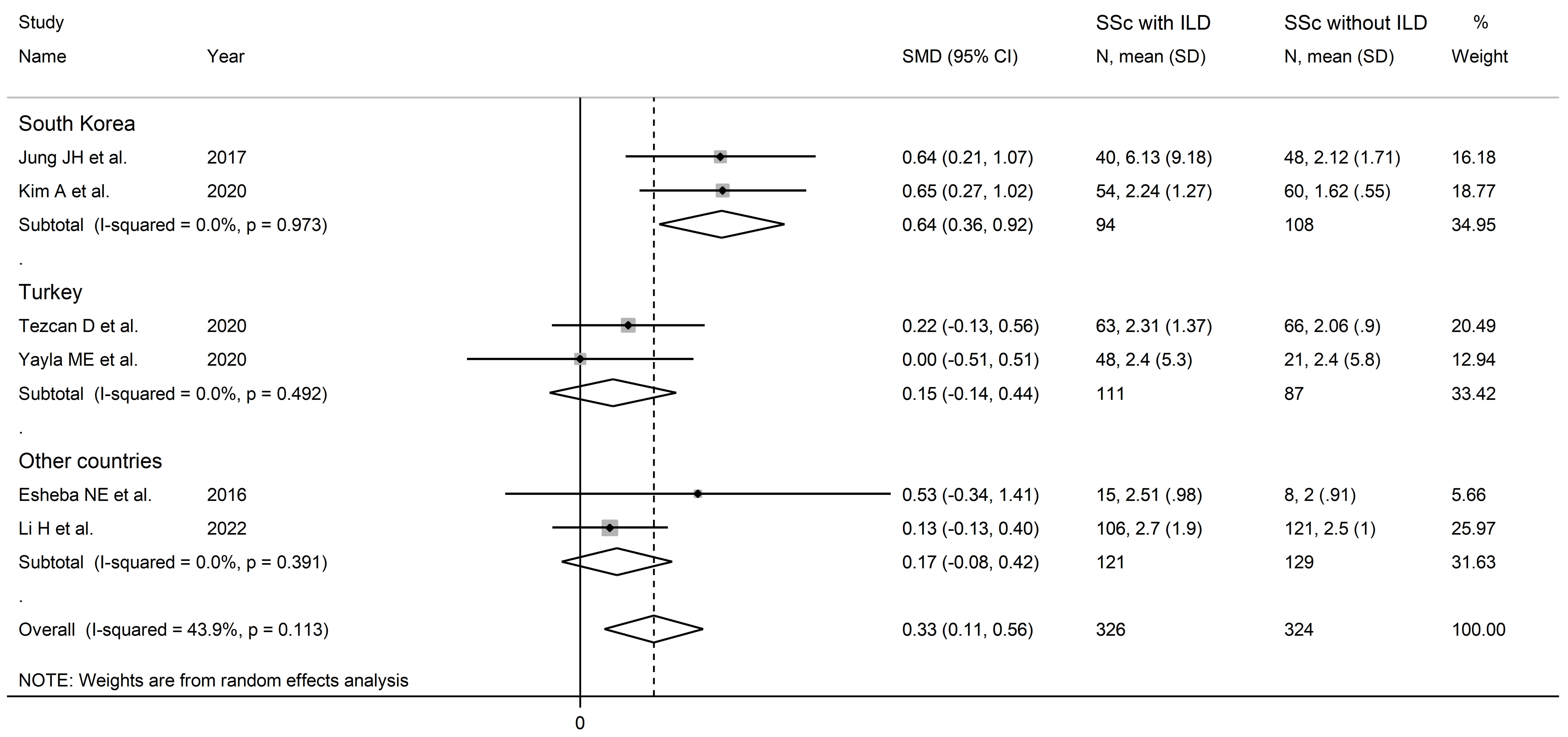

Supplement: Supplementary file 9 [file Image_8.tif]
