# Supplementary material for: The association between the neutrophil-to-lymphocyte ratio, platelet-to-lymphocyte ratio, and monocyte-to-lymphocyte ratio and systemic sclerosis and its complications: a systematic review and meta-analysis
Source: Front Immunol. 2024 May 10;15:1395993. doi: 10.3389/fimmu.2024.1395993 (PMC11116674; doi:10.3389/fimmu.2024.1395993)
Supplement: Supplementary file 11 [file Table_2.docx]

**Supplementary Table 2.** Assessment of the risk of bias using the Joanna Briggs Institute critical appraisal checklist.

| **Study** | **Were the inclusion criteria clearly defined?** | **Were the subjects and the setting described in detail?** | **Was the exposure measured in a reliable way?** | **Were standard criteria used to assess the condition?** | **Were confounding factors identified?** | **Were strategies to deal with confounding factors stated?** | **Were the outcomes measured in a reliable way?** | **Was appropriate statistical analysis used?** | **Risk of bias** |
| --- | --- | --- | --- | --- | --- | --- | --- | --- | --- |
| Esheba NE et al. (1) | Yes | Yes | Yes | Yes | No | No | Yes | Yes | Low |
| Yolbas S et al. (2) | Yes | Yes | Yes | Yes | No | No | Yes | Yes | Low |
| Yang Z et al. (3) | Yes | Yes | Yes | Yes | No | No | Yes | Yes | Low |
| Jung JH et al. (4) | Yes | Yes | Yes | Yes | No | No | Yes | Yes | Low |
| Kim A et al. (5) | Yes | Yes | Yes | Yes | Yes | Yes | Yes | Yes | Low |
| Tezcan D et al. (6) | Yes | Yes | Yes | Yes | No | No | Yes | Yes | Low |
| Yayla ME et al. (7) | Yes | Yes | Yes | Yes | Yes | Yes | Yes | Yes | Low |
| Sakr BR et al. (8) | Yes | Yes | Yes | Yes | No | No | Yes | Yes | Low |
| Li H et al. (9) | Yes | Yes | Yes | Yes | Yes | Yes | Yes | Yes | Low |
| Nejatifar F et al. (10) | Yes | Yes | Yes | Yes | Yes | Yes | Yes | Yes | Low |

**References**

1. Esheba NE, Shahba A. Assessment of Neutrophil Lymphocyte Ratio in Systemic Sclerosis Patients in Tanta University Hospital: A Promising Marker in Predicting Disease Severity. Egypt J Rheumatol Clin Immunol. 2016;4:43-7. doi:

2. Yolbas S, Yildirim A, Gozel N, Uz B, Koca SS. Hematological Indices May Be Useful in the Diagnosis of Systemic Lupus Erythematosus and in Determining Disease Activity in Behcet's Disease. Med Princ Pract. 2016;25(6):510-6. doi: 10.1159/000447948

3. Yang Z, Zhang Z, Lin F, Ren Y, Liu D, Zhong R, Liang Y. Comparisons of neutrophil-, monocyte-, eosinophil-, and basophil- lymphocyte ratios among various systemic autoimmune rheumatic diseases. APMIS. 2017;125(10):863-71. doi: 10.1111/apm.12722

4. Jung J-H, Lee Y-M, Lee E-G, Yoo W-H, Lee W-S. Neutrophil-to-lymphocyte Ratio in Diagnosis of Systemic Sclerosis for Prediction of Interstitial Lung Disease. Journal of Rheumatic Diseases. 2017;24(3). doi: 10.4078/jrd.2017.24.3.138

5. Kim A, Kim Y, Kim GT, Ahn E, So MW, Sohn DH, Lee SG. Platelet-to-lymphocyte ratio and neutrophil-to-lymphocyte ratio as potential makers for digital ulcers and interstitial lung disease in patients with systemic sclerosis: cross-sectional analysis of data from a prospective cohort study. Rheumatol Int. 2020;40(7):1071-9. doi: 10.1007/s00296-020-04604-6

6. Tezcan D, Turan Ç, Yılmaz S, Sivrikaya A, Gülcemal S, Limon M, Ecer B. What do simple hematological parameters tell us in patients with systemic sclerosis? Acta Dermatovenerologica Alpina Pannonica et Adriatica. 2020;29(3). doi: 10.15570/actaapa.2020.23

7. Yayla ME, Ilgen U, Okatan IE, UsluYurteri E, Torgutalp M, Kelesoglu Dincer AB, et al. Association of simple hematological parameters with disease manifestations, activity, and severity in patients with systemic sclerosis. Clin Rheumatol. 2020;39(1):77-83. doi: 10.1007/s10067-019-04685-0

8. Sakr BR, Rabea RE, ElHamid SM. Value of hematological parameters as biomarkers of disease manifestations and severity in systemic sclerosis. The Egyptian Rheumatologist. 2021;43(2):159-65. doi: 10.1016/j.ejr.2020.06.008

9. Li H, Zhang X, Yu L, Shang J, Fan J, Feng X, et al. Comparing clinical characteristics of systemic sclerosis with or without interstitial lung disease: A cross-sectional study from a single center of the Chinese Rheumatism Data Center. Front Med (Lausanne). 2022;9:1061738. doi: 10.3389/fmed.2022.1061738

10. Nejatifar F, Mirbolouk N, Masooleh IS, Kazemnejad E, Ghavidel-Parsa B, Ghanbari AM, Zayeni H. Association between neutrophil/lymphocyte ratio and disease severity in scleroderma patients. Heliyon. 2023;9(10):e20576. doi: 10.1016/j.heliyon.2023.e20576
